# Pulmonary metastasectomy for malignant melanoma prolongs survival significantly compared to non-surgical therapy

**DOI:** 10.1007/s11748-026-02310-x

**Published:** 2026-06-01

**Authors:** Georg Schlachtenberger, Abraham Goitom, Andres Amorin, Alberto Lopez Pastorini, Julika Merres, Christopher Gaisendrees, Matthias B. Heldwein

**Affiliations:** 1 Department of Thoracic Surgery, Hildegardis Hospital Cologne, Cologne, Germany; 2 Faculty of Medicine, University of Cologne, Bachemer Str. 29-33, 50931 Cologne, Germany; 3 School of Medicine, University of Cologne, Cologne, Germany; 4 Department of General, Visceral, Thoracic and Transplant Surgery, Faculty of Medicine, University Hospital Cologne, Cologne, Germany

**Keywords:** Pulmonary metastasectomy, Isolated pulmonary metastases, Melanoma, Long-term survival

## Abstract

**Objectives:**

Pulmonary metastasectomy (PM) is an established treatment for selected patients with pulmonary metastases. While systemic therapies such as immune checkpoint inhibitors, and stereotactic radiation have become standard for unresectable or metastasized melanoma patients, no randomized controlled trials directly assess the benefit of PM by itself in oligometastatic melanoma patients with lung metastases. This retrospective study compares long-term survival outcomes in patients undergoing PM versus those receiving non-surgical therapy for pulmonary metastatic melanoma.

**Methods:**

We retrospectively analyzed melanoma patients from the skin cancer center Cologne who were treated with curative intent for their primary tumor and later developed isolated pulmonary metastases. Patients in the non-surgical group had biopsy-confirmed lung metastases. Survival outcomes between patients undergoing PM and those receiving non-surgical therapies were compared.

**Results:**

Between 2012 and 2020, 87 patients met the inclusion criteria: 49 underwent PM and 38 received non-surgical therapy. The number of pulmonary metastases differed significantly between groups (1.27 ± 0.57 vs. 3.2 ± 1.1; *p* < 0.001). BRAF-mutations were more common in the non-surgical group (35.4% vs. 55.3%; *p* = 0.04).

Patients undergoing PM showed significantly improved 3-year (88.0% vs. 65.7%; *p* = 0.04) and 5-year survival rates (81.3% vs. 30.8%; *p* = 0.002) compared to the non-surgical group. Kaplan–Meier analysis confirmed prolonged overall survival in the PM group (log-rank *p* < 0.02). However, among patients with BRAF mutations, survival did not differ significantly between the groups (*p* = 0.09).

**Conclusions:**

PM is associated with favorable overall survival. These findings support considering PM as a treatment option in selected patients to improve long-term outcomes.

**Graphical abstract:**

**Figure:**
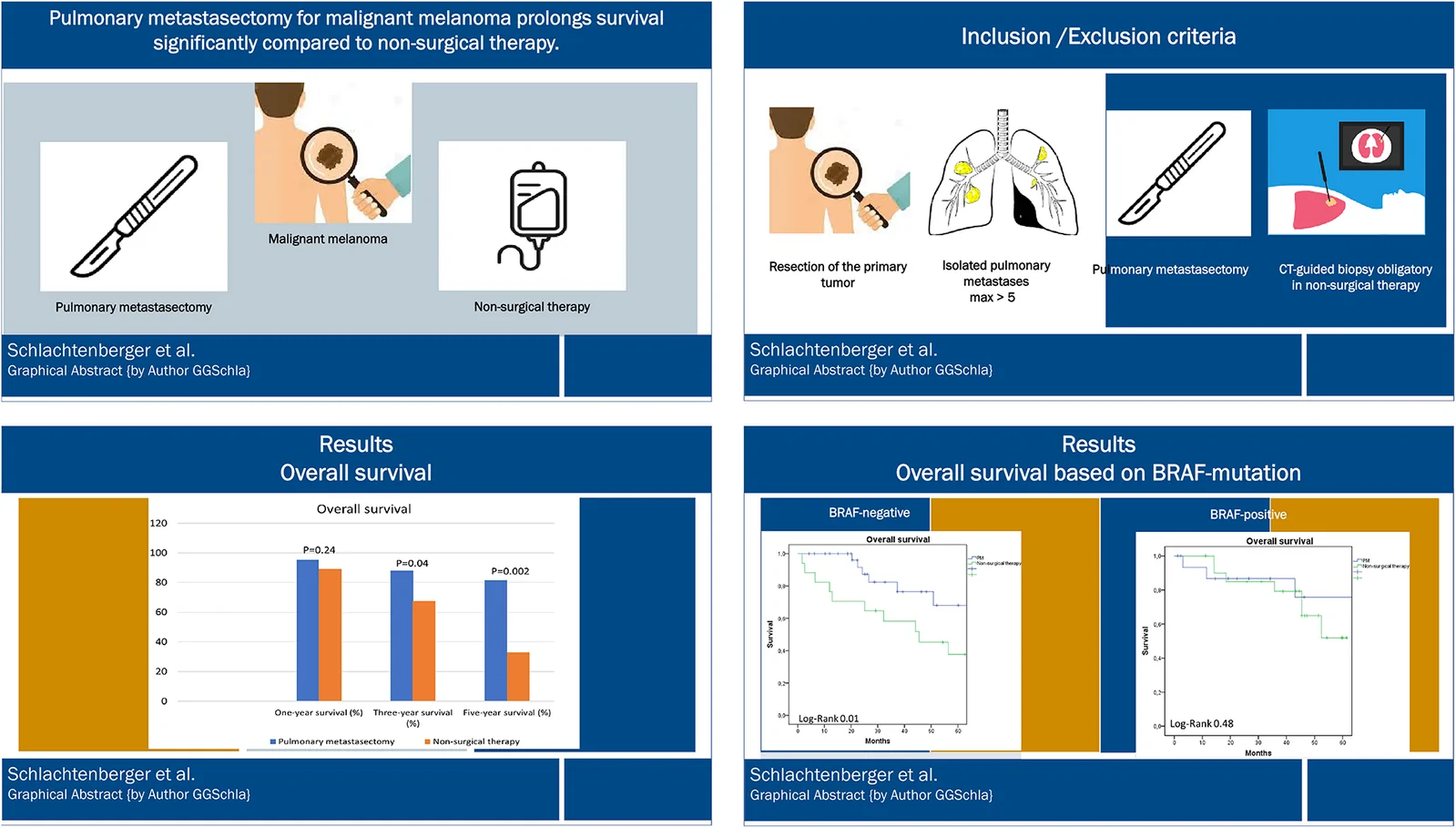

## Introduction

Malignant melanoma is a cancer originating from melanocytes in the epidermis, with increasing incidence rates, particularly among Caucasians. Cutaneous melanoma is primarily associated with ultraviolet (UV) radiation exposure from natural sunlight and indoor tanning; however, several melanoma subtypes arise independently of UV exposure [1]. Early-stage disease, such as stage I melanoma, carries a favorable prognosis, with 5-year overall survival (OS) rates exceeding 90% [2]. The lungs represent the most frequent site of distant metastasis, being affected by over 30% of patients with stage IV melanoma [3, 4]. Historically, metastatic melanoma was associated with poor outcomes due to the limited effectiveness of chemotherapy, resulting in 5-year survival rates of less than 10% [5, 6]. Pulmonary metastasectomy (PM), a surgical procedure performed with curative intent in carefully selected patients with limited disease burden and resectable metastases, has been shown to improve survival outcomes. [7–9]. In these cases, median survival times of up to 18.3 months and 5-year survival rates approaching 35% have been reported [3, 7, 10]. In recent years, systemic therapies have markedly evolved, particularly with the introduction of targeted therapies (e.g., BRAF and MEK inhibitors), incorporated into European guidelines since 2012, and immune checkpoint inhibitors (e.g., PD-1 and CTLA-4 inhibitors), included since 2011, significantly improving survival outcomes in patients with metastatic melanoma. Contemporary 5-year survival rates with these agents now reach approximately 34% [4, 11, 12]. Given these advances, the role of PM in the management of pulmonary metastatic melanoma is being reconsidered. Despite its potential benefits, PM remains underutilized and is largely absent from current melanoma treatment guidelines. This may be due in part to limited awareness among dermatologists and the absence of randomized controlled trials (RCTs) directly comparing PM to non-surgical treatments [13, 14]. Furthermore, no retrospective studies have previously compared survival outcomes between these two treatment strategies.

This study aimed to evaluate overall survival in patients with isolated pulmonary metastatic malignant melanoma by treatment modality. Specifically, we retrospectively compared long-term survival outcomes between patients who underwent pulmonary metastasectomy as first treatment for isolated melanoma lung metastases and those who received non-surgical therapies.

## Methods

### Ethic statement

This study was approved by the Institutional Review Board of the University Hospital of Cologne and conducted in accordance with the principles outlined in the Declaration of Helsinki. Due to the retrospective nature of the analysis, the requirement for written informed consent was waived on September 15, 2022.

All patients diagnosed with metastatic melanoma and treated at the skin cancer center of the University Hospital of Cologne between January 2012, and December 2020 were included in this retrospective analysis. Medical records were reviewed, and relevant clinical, therapeutic, and follow-up data were extracted from the institutional patient database. Collected variables included perioperative details, systemic therapy administration (chemotherapy, immunotherapy, and checkpoint inhibitors), and long-term survival outcomes.

### Inclusion and exclusion criteria

Patients were eligible for inclusion only if they had only pulmonary metastases and no affection of other organ structures. Additionally, the primary melanoma must have been treated with curative intent. Patients with 1–5 pulmonary metastases were included. In the non-surgical therapy group, inclusion required histological confirmation that radiologically suspected metastases originated from the corresponding melanoma, typically obtained through CT-guided biopsy. The interval from diagnosis—established by CT-guided biopsy and subsequent histological examination—to treatment initiation in the non-surgical group was minimal, though a short delay was inevitably present.

These stringent inclusion and exclusion criteria were established to ensure a well-matched patient population across both treatment arms, thereby minimizing confounding variables and allowing for a more accurate comparison of long-term survival outcomes.

### Diagnosis and treatment

The attending dermatologist initially evaluated patients with melanoma and suspected newly emerged pulmonary metastases. Positron Emission Tomography/Computed Tomography (PET-CT) was performed in patients with suspected distant metastases [14].

Surgical resection was considered only for patients with adequate cardiopulmonary fitness and when an R0 resection was highly achievable.

In cases where patients declined surgery or when metastases were located in positions requiring extensive lung resections (e.g., bilobectomy or pneumonectomy) to achieve R0 resection, alternative treatments such as chemotherapy, immunotherapy, radiation therapy, or checkpoint inhibitors were administered.

The most common surgical approach for pulmonary metastases was wedge resection performed via uniportal video-assisted thoracic surgery (VATS). Lymphadenectomy was performed only in cases where CT/PET-CT imaging indicated suspected mediastinal lymphadenopathy.

In cases of bilateral pulmonary metastases, surgical management was consistently performed in two stages to minimize perioperative risk and allow adequate recovery between procedures.

In the non-surgical group, all patients were treated in accordance with melanoma guidelines. As targeted therapies (BRAF and MEK inhibitors) and immune checkpoint inhibitors (PD-1 and CTLA-4 inhibitors) were incorporated into European guidelines in 2012 and 2011, respectively, both were available throughout the study period [13–15].

### Statistical analysis

Follow-up data were obtained from our institutional patient database. One-year, three-year, and five-year survival rates were calculated for the pulmonary metastasis (PM) group, measured from the time of surgery to the date of death. In the non-surgical therapy group, survival rates were measured from the time of definitive pathological diagnosis of pulmonary metastases to the initiation of non-surgical therapy, and from that point until death. The mean overall survival following PM surgery or the diagnosis and treatment of pulmonary metastases in the non-surgical therapy group was assessed using Kaplan–Meier curves and compared using the log-rank test. Cox regression model was assessed for multivariable survival analysis described by hazard ratio (HR) and 95% confidence interval (CI). The disease-free interval (DFI) was also measured, defined as the duration between the surgery for the primary tumor and the onset of pulmonary metastases. Categorical variables were analyzed using Pearson’s chi-squared or Fisher’s exact test. Continuous variables were expressed as mean ± standard deviation (SD) and analyzed using an unpaired Student’s t-test. Additionally, a multinomial logistic regression analysis was performed, including age at the time of metastasis diagnosis and the number of metastases as covariates. Differences were reported as odds ratios (ORs) with 95% confidence intervals (CIs). A *p*-value of < 0.05 was considered statistically significant.

## Results

**Fig. 1:**
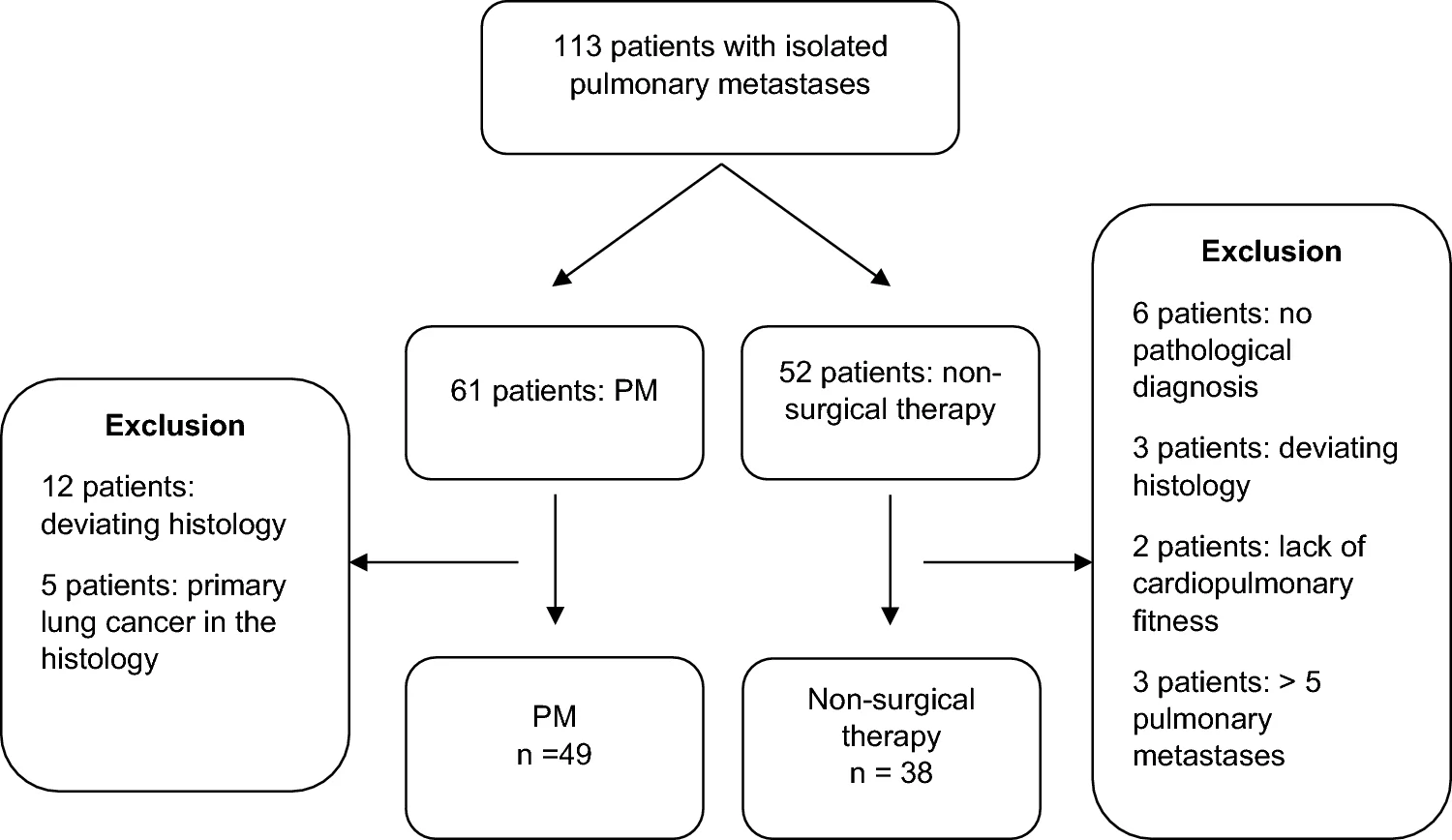
Flow chart concerning the inclusion and exclusion criteria. PM: pulmonary metastasectomy

Between January 2012 and December 2020, the attending dermatologist presented 131 patients with isolated pulmonary metastases at our multidisciplinary team (MDT) meetings. The mean follow-up duration for the cohort was 40.8 ± 27.2 months. Figure 1 outlines the patients excluded from further analysis based on the applied inclusion and exclusion criteria. Ultimately, 87 patients (66.4%) met all criteria and were included in the analysis.

Of these, 49 patients underwent PM, while 38 received non-surgical therapy.

### Primary malignant melanoma

Table 1 summarizes the data on primary malignant melanoma. The mean age of patients undergoing surgery for primary melanoma was 58.7 ± 14.7 years, with no significant age difference observed between the groups. The gender distribution was similar, with 40.6% females and 60.4% males (*p* = 0.46). The majority of primary melanomas were superficial spreading melanomas (31.0%), followed by nodular melanoma (27.6%), amelanotic melanoma (9.2%), and lentigo melanoma (6.9%). Histological analyses showed no significant differences between groups.

**Table 1:** Primary malignant melanoma

	Total cohort n = 87	PM n = 49	Non-surgical therapy n = 38	*p*-value
Age of primary Melanoma surgery (years)	58.7 ± 14.7	54.2 ± 15.2	57.9 ± 11.2	0.28
Male gender (%)	63.2	64.6	60.4	0.46
Primary tumor thickness (mm)	4.5 ± 5.0	4.1 ± 5.7	4.9 ± 3.8	0.45
*N-state primary tumor:*				
pN0 (%)	14.9	16.3	13.2	0.46
pN1 (%)	37.9	44.9	28.9	0.18
pN2 (%)	27.6	24.5	31.6	0.31
pN3 (%)	19.5	14.3	26.3	0.14
*Stage primary tumor*				
Stage 1 (%)	2.2	2.1	2.4	0.91
Stage 2 (%)	16.1	16.7	15.3	0.65
Stage 3 (%)	49.5	50.1	48.2	0.57
Stage 4 (%)	32.6	31.1	34.1	0.41
*Histology primary tumor:*				
Amelanotic melanoma(%)	9.2	10.2	7.9	0.13
Lentigo Melanoma (%)	6.9	8.2	5.3	0.67
Nodular Melanoma(%)	27.6	26.5	28.9	0.83
Superficial spreading melanoma(%)	31.0	32.7	28.9	0.12
Adjuvant therapy primary tumor				
Chemo/Imunotherapy	65.5	38.8	97.4	**< 0.001**
Radiation therapy	15.1	12.2	18.4	0.55

Abbreviations: PM: pulmonary metastasectomy

statistically significant p-values are indicated in bold

Nodal stage of the primary tumor was analyzed, but no significant differences were found between groups: pN0 (16.3% vs. 13.2%, *p* = 0.46), pN1 (44.9% vs. 28.9%, *p* = 0.18), pN2 (24.5% vs. 31.6%, *p* = 0.31), and pN3 (14.3% vs. 26.3%, *p* = 0.14). Similarly, tumor stage did not differ significantly between groups. Overall, tumor stage also did not differ significantly between groups.

### Baseline characteristics of pulmonary metastases

Baseline characteristics of pulmonary metastases are detailed in Table 2. General cardiopulmonary fitness was assessed using the American Society of Anesthesiologists (ASA) classification system, which revealed no significant differences between groups; the majority of patients were classified as ASA I (61.2% vs. 68.4%, *p* = 0.02). Among the metastases, 27.1% were located in the left lung (26.8% vs. 28.6%, *p* = 0.79), and 43.2% were in the right lung (46.5% vs. 39.1%, *p* = 0.10). There was no significant difference in the occurrence of bipulmonary metastases (29.7% vs. 26.7%, *p* = 0.13).

**Table 2:** Baseline characteristics of pulmonary metastases

	Total cohort n = 87	PM n = 49	Non-surgical therapy n = 38	*p*-value
Age at diagnosis of metastases (years)	64.2 ± 15,1	64.9 ± 14,1	63.1 ± 16.5	0.58
Lesion size (cm) (biggest)	1.32 ± 1.1	1.49 ± 1.36	1.11 ± 0.78	0.12
Number of metastases (n.)	2.11 ± 1.3	1.67 ± 0.57	2.8 ± 1.1	**0.02**
*Localisation of the metastases:*				
Left (%)	27.1	26.8	28.6	0.79
Right (%)	43.2	46.5	39.1	0.10
Bipulmonary (%)	29.7	26.7	32.3	0.13
DFI (months)	37.3 ± 17.1	39.7 ± 17.6	33.6 ± 16.2	0.21
*ASA:*				
ASA I (%)	64.8	61.2	68.4	**0.02**
ASA II (%)	31.8	32.7	31.6	**0.03**
ASA III (%)	3.4	6,1	0	n.s
*ECOG:*				
ECOG 0 (%)	76.5	79.1	73.7	0.24
ECOG 1 (%)	19.8	16.3	23.7	0.67
ECOG 2 (%)	3.5	4.7	2.6	0.39

ASA classification: American Society of Anesthesiology classification; ECOG: Eastern Cooperative Oncology Group; DFI: Disease-free interval; PM: Pulmonary metastasectomy

statistically significant p-values are indicated in bold

The number of metastases differed significantly between groups (1.67 ± 0.6 vs. 2.8 ± 1.1, *p* = 0.02). Lesion size of the biggest metastasis did not differ significantly between the groups (1.6 ± 1.1 cm vs. 1.7 ± 0.9 cm, *p* = 0.50). The mean age of patients diagnosed with pulmonary metastases was 59.1 ± 12.1 years, with no significant age difference between groups.

The disease-free interval (DFI) from primary tumor treatment to the appearance of the first pulmonary metastasis was 37.3 ± 17.1 months. The DFI did not differ between groups.

### Pulmonary metastases therapy

Table 3 presents data on the therapy of pulmonary metastases. Wedge resection was the most common approach among patients undergoing surgery, performed in 84.0% of cases. Segmentectomy was performed in 4.0%, and lobectomy in 12.0% of patients. R0 resection was achieved in 100% of surgical patients. Postoperative complications included thoracotomy for pleural fistula in 1.2% and pneumonia requiring intravenous antibiotics in 4.1% of patients. The mean postoperative in-hospital stay was 4.5 ± 1.7 days, with no hospital readmissions reported within 90 days. The 30-day mortality rate was 0%. Notably, 36% of patients in the surgical group received perioperative immunotherapy as part of an oligometastatic treatment strategy. In the non-surgical therapy group, 18.4% of patients received radiation therapy, while 81.6% underwent immune or checkpoint inhibitor therapy. Of these, 39.5% received pembrolizumab, 32.4% received nivolumab, 10.5% received ipilimumab, and 7.9% received ipilimumab + nivolumab. Additionally, 15.8% were treated with encorafenib + binimetinib, and 5.3% with dabrafenib + trametinib. Therapy was interrupted or discontinued in 18.4% of patients due to adverse effects. In the multinomial logistic regression analysis comparing pulmonary metastasectomy (PM) and non-surgical therapy (Table 4), no significant associations were found for age at the time of metastasis diagnosis (OR 1.04, 95% CI: 0.98–1.01; *p* = 0.28), BRAF status (OR 2.28, 95% CI: 0.38–13.54; *p* = 0.37), or ECOG status (OR 1.85, 95% CI: 0.26–12.22; *p* = 0.51). However, the number of metastases was significantly higher in the non-surgical therapy group (OR 8.66, 95% CI: 3.08–24.32; *p* < 0.001).

**Table 3:** Pulmonary metastases therapy

	Total cohort n = 87	PM n = 49	Non-surgical therapy n = 38	*p*-value
*Pulmonary metastasectomy:*				
VATS/Thoracotomy		76.2/23.8		
Wedge resection (%)		84.0		
Segmentectomy (%)		4.0		
Lobectomy (%)		12.0		
Perioperative complications (%)		5.2		
In-hospital stay (days)		4.5 ± 1.7		
30-day mortality (%)		0		
Perioperative immunotherapy (%)		38		
*Non-surgical therapy:*				
Nivolumab (%)			10.5	
Ipilimumab (%)			7.9	
Pembrolizumab (%)			39.5	
Ipilimumab + Nivolumab (%)			21.2	
Encorafenib + Binimetinib (%)			15.8	
Dabrafenib + Trametinib (%)			5.3	
Radiation (%)			18.4	
Interrupt or discontinue of therapy(%)			18,4	
BRAF positive (%)	44.2	35.4	55.3	**0.04**
PDL1 positive (%)	60.5	60.0	60.6	0.81

BRAF: B-Raf proto-oncogene, serine/threonine kinase, PM: Pulmonary metastasectomy; PDL1: Programmed Death-Ligand 1; VATS: Video-assisted thoracic surgery

statistically significant p-values are indicated in bold

**Table 4:** Multinomial regression analysis for PM versus non-surgical therapy

Variable	OR	CI 95%	*p*-value
Age at time diagnosis of the metastases	1.04	0.98–1.01	0.26
Number of Lung metastases	6.66	3.08–19.32	0.05
BRAF-Status:	2.26	0.38–13.54	0.37
ECOG	1.85	0.26–12.22	0.52

BRAF: B-Raf proto-oncogene, serine/threonine kinase, CI: confidence interval, ECOG: Eastern Cooperative Oncology Group, OR: odds ratio

The 90-day hospital readmission rate for patients in the non-surgical therapy group was 12.3%. A BRAF mutation was identified in 44.2% of patients, with a significantly higher proportion in the non-surgical group (55.3%) compared to the PM group (35.4%, *p* = 0.04). PDL-1 expression was evenly distributed between the two groups (*p* = 0.81). 17.2% of patients received a combination of PM and adjuvant treatment.

### Overall survival

Table 5a, b, and c present the one-year, three-year, and five-year survival rates for the overall groups and the subgroups stratified by BRAF mutation status. Among all patients, the one-year survival rate for those undergoing pulmonary metastasectomy (PM) was 95.5%, compared to 89.2% for those receiving non-surgical therapy (*p* = 0.24). At three years, 88% of PM patients were alive, compared to 67.6% of non-surgical therapy patients (*p* = 0.04). The five-year survival rate was significantly higher for PM patients at 53.4%, compared to 20.0% for non-surgical therapy patients (*p* = 0.02).

**Table 5:** **a** Overall survival. **b** Overall survival BRAF-negative. **c** Overall survival BRAF-positive

(a)	Total cohort n = 87	PM n = 49	Non-surgical therapy n = 38	*p*-value
One-year survival (%)	92.8	95.6	89.5	0.26
Three-year survival (%)	59.7	71.4	50.0	0.007
Five-year survival (%)	44.7	61.9	30.8	0.003

(b)	Total cohort n = 49	PM n = 32	Non-surgical therapy n = 17	*p*-value
One-year survival (%)	91.5	100	76.5	0.01
Three-year survival (%)	67.6	77.8	43.8	0.008
Five-year survival (%)	46.4	68.0	38.2	0.01

(c)	Total cohort n = 38	PM n = 17	Non-surgical therapy n = 21	*p*-value
One-year survival (%)	94.4	86.7	100	0.17
Three-year survival (%)	82.8	80.0	83.3	0.64
Five-year survival (%)	52.6	60.0	51.4	0.17

BRAF: B-Raf proto-oncogene, serine/threonine kinase, PM: Pulmonary metastasectomy

Subgroup analysis revealed differing outcomes based on BRAF mutation status. Among the 38 patients with a BRAF mutation (Table 5b), including 17 undergoing PM and 21 receiving non-surgical therapy, survival rates did not significantly differ between groups (one-year: 86.7% vs. 100%, *p* = 0.17; three-year: 80.0% vs. 83.3%, *p* = 0.64; five-year: 60.0% vs. 51.4%, *p* = 0.17). In contrast, among the 49 patients without a BRAF mutation (Table 5c), including 32 undergoing PM and 17 receiving non-surgical therapy, survival rates were significantly better in the PM group (one-year: 100% vs. 76.5%, *p* = 0.01; three-year: 77.8% vs. 43.8%, *p* = 0.008; five-year: 68.0% vs. 38.2%, *p* = 0.01).

**Fig. 2:**
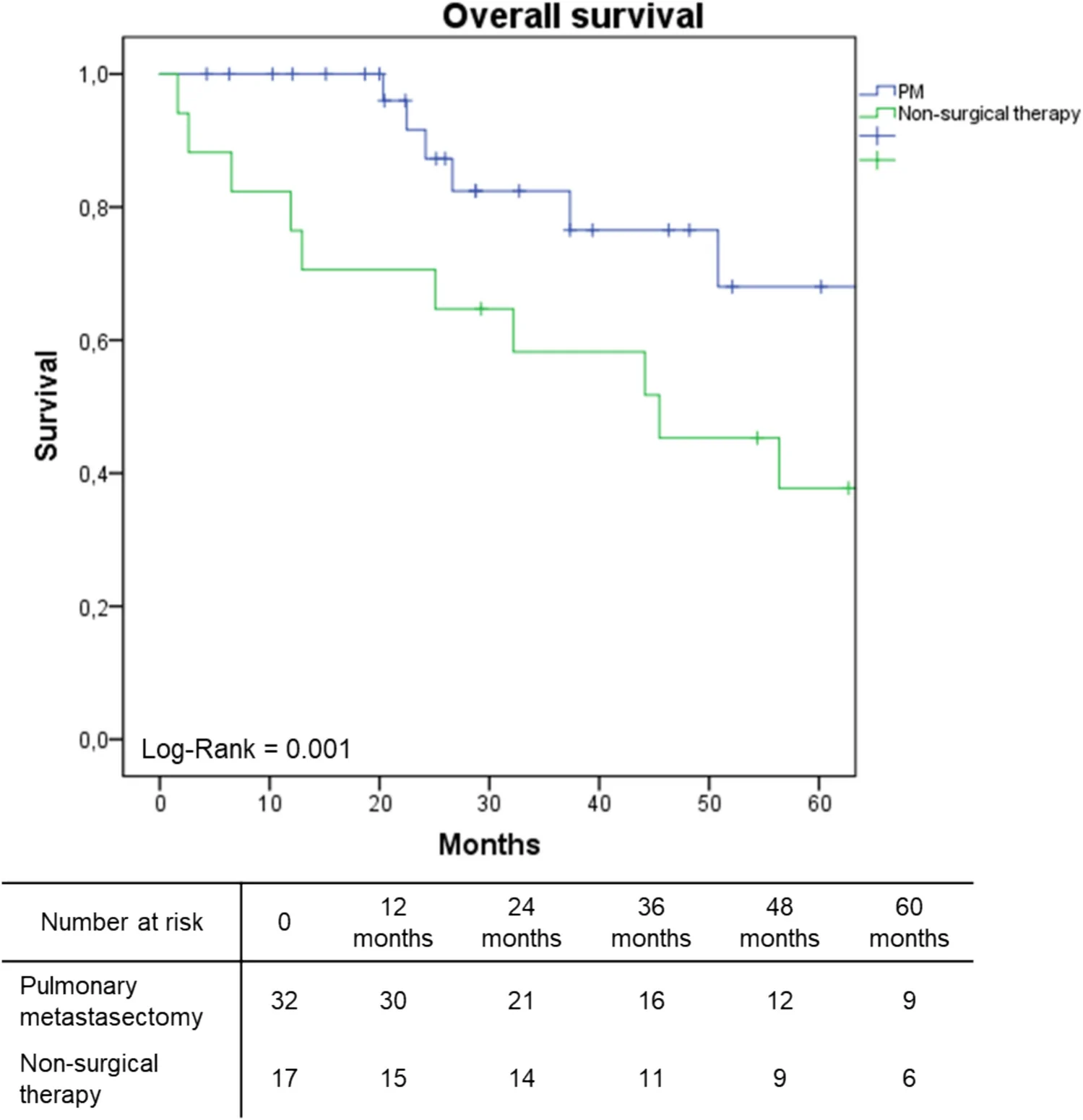
Kaplan–Meier plot for overall survival months after surgery in the PM group and from the time of definitive pathological diagnosis of pulmonary metastases and the consecutive first administration of any non-surgical treatment in the non-surgical therapy group

Figure 2 shows the overall survival for all patients. Those who underwent PM had a significantly prolonged survival compared to non-surgical therapy patients (log-rank *p* = 0.02).

**Fig. 3:**
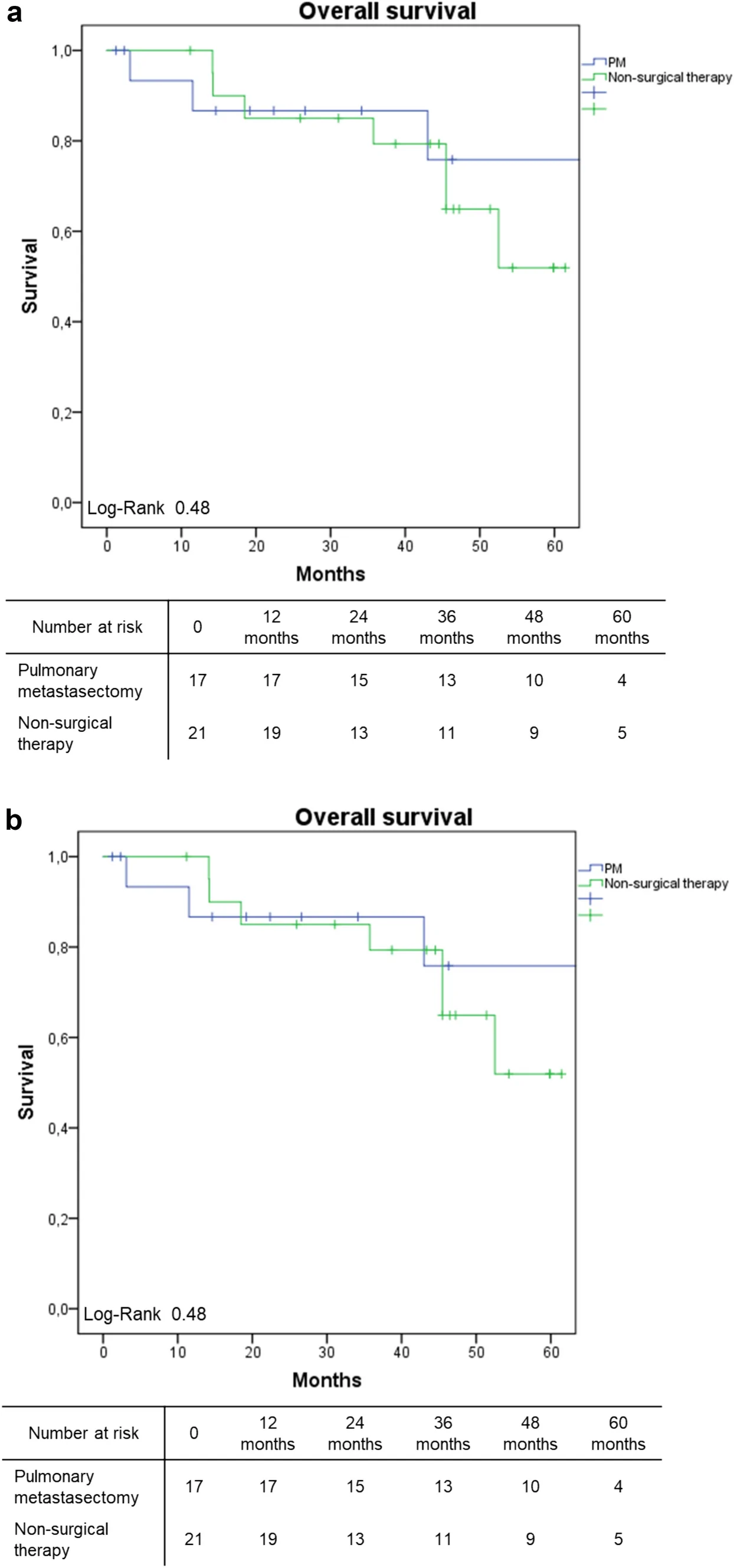
**a, b:** Kaplan–Meier plot for overall survival months after surgery in the PM group and from the time of definitive pathological diagnosis of pulmonary metastases and the consecutive first administration of any non-surgical treatment in the non-surgical therapy group. Stratified by BRAF mutation status. **a** focuses on patients with BRAF mutation. **b** shows survival for patients with a BRAF wildtype

Figure 3a and b illustrate Kaplan–Meier survival curves comparing outcomes between patients undergoing PM and those receiving non-surgical therapy, stratified by BRAF mutation status. Figure 3a focuses on patients with BRAF mutation. In this subgroup, no significant difference in survival was observed between the PM and non-surgical therapy groups (log-rank *p* = 0.48), suggesting comparable effectiveness of targeted therapies and surgical intervention.

Figure 3b shows survival for patients with a BRAF wildtype. Here, survival was significantly better in the PM group (log-rank *p* = 0.02).

**Fig. 4:**
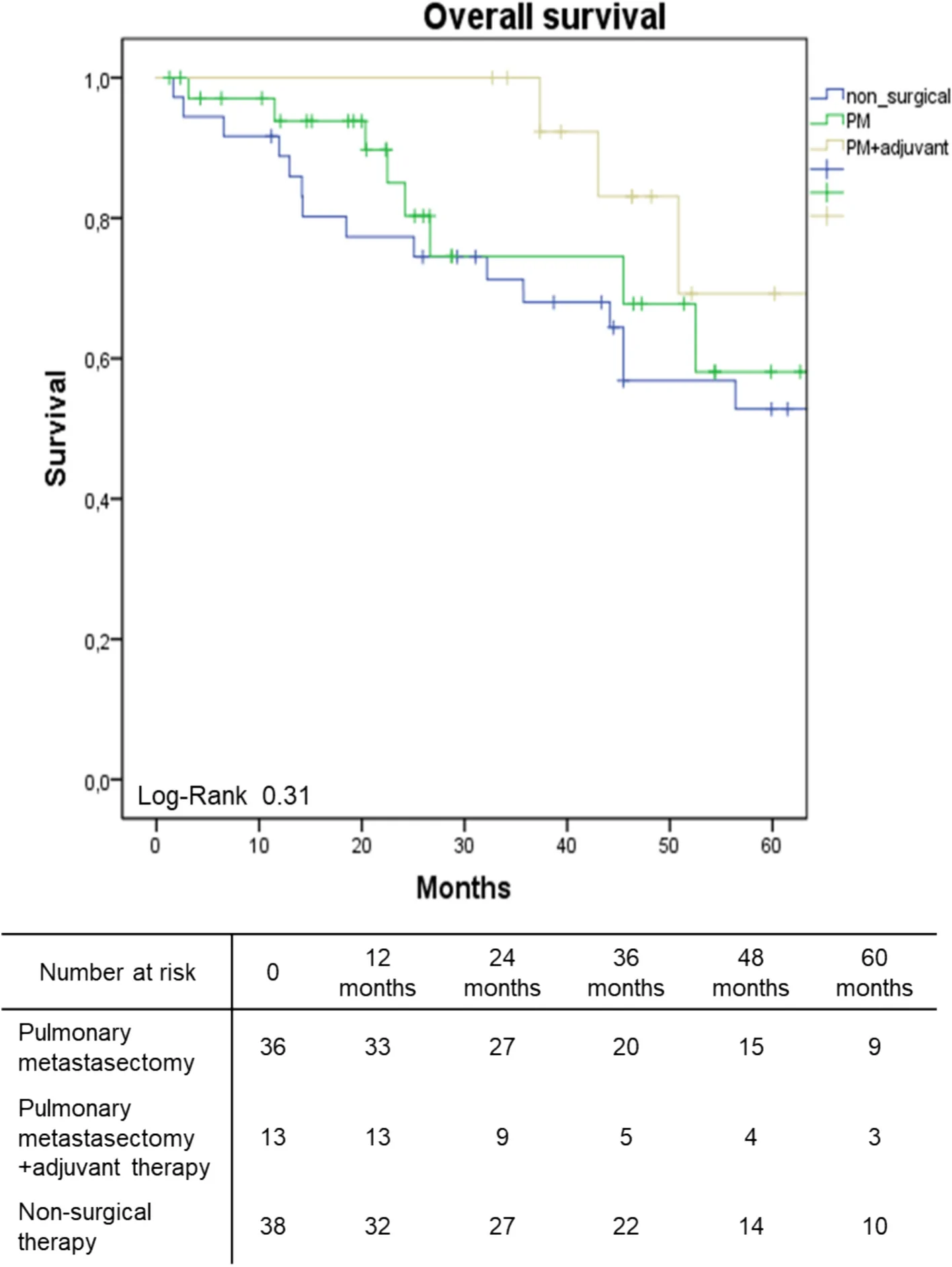
Kaplan–Meier plot for overall survival months after surgery in the PM and PM + adjuvant therapy group and from the time of definitive pathological diagnosis of pulmonary metastases and the consecutive first administration of any non-surgical treatment in the non-surgical therapy group

Figure 4 illustrates the survival of patients who underwent pulmonary metastasectomy (PM) with adjuvant therapy and PM alone, compared to those who received non-surgical therapy (log-rank 0.31).

These survival curves visually reinforce the findings from Fig. 4 and support the selective use of PM, particularly in patients with BRAF wildtype. Cox regression analysis showed that the BRAF-Status and the therapeutic regime significantly influenced the overall survival rate (HR 4.96, CI 1.81–30.18; *p* = 0.008), (HR 2.99 CI 1.12–8.03; *p* = 0.03). We saw no significant influence of DFI (HR 0.98 CI 0.98–1.0; *p* = 0.52), or the number of metastases (HR 1.53, CI 0.85–2.76; *p* = 0.15) on overall survival (Table 6).

**Table 6:** Cox regression analysis for overall survival

Variable	HR	CI 95%	*p*-value
BRAF-Status	4.96	1.81–30.18	**0.008**
PDL-1 Status:	0.89	0.62–1.27	0.50
PM/Non-surgical therapy:	2.99	1.12–8.03	**0.03**
Bilateral metastases:	1.59	0.83–1.48	0.31
DFI:	0.98	0.98–1,04	0.52
N-Status of the primary tumor:	1.51	0.73–3.16	0.27
LDH at the time of diagnosis of metastases:	1.01	0.99–1.02	0.10
Number of metastases:	1.53	0.72–2.71	0.54
Size of the metastases (cm)	1.63	0.85–2,76	0.15

BRAF: B-Raf proto-oncogene, serine/threonine kinase, CI: confidence interval, DFI: disease free intervall, HR: Harzard ratio, LDH: Lactate dehydrogenase, PDL-1: Programmed Cell Death-Ligand 1, PM: pulmonary metastasectomy

statistically significant p-values are indicated in bold

## Discussion

This retrospective two-arm study on the treatment of patients with malignant melanoma and isolated pulmonary metastases demonstrated significantly better long-term survival rates for those undergoing pulmonary metastasectomy (PM) compared to patients receiving non-surgical therapy. To our knowledge, this is the first retrospective parallel-arm study to compare these.

Patients with a BRAF mutation showed similar survival rates between PM and non-surgical therapy. The most common surgical approach was VATS wedge resection, performed in 76.2% of patients, with an R0 resection achieved in 100% of cases. Conversely, 18.4% of patients in the non-surgical therapy group had to interrupt or discontinue treatment due to severe side effects.

The average hospital stay for patients undergoing PM was 4.5 ± 1.7 days. The 30-day mortality rate was 0%, and no hospital readmissions occurred within 90 days post-discharge, suggesting that PM is a safe procedure.

### Are RCTs to be expected?

Although PM is widely accepted as potentially curative in selected cases of colorectal carcinoma, sarcoma, and malignant melanoma, this acceptance persists despite the absence of robust randomized controlled trial (RCT) data [16]. The only RCT conducted to date, by Treasure et al., was terminated prematurely in 2019 due to insufficient patient enrollment. The study aimed to compare active surveillance with PM in patients with pulmonary metastatic colorectal carcinoma. Initially, the authors planned to enroll 1500 patients, aiming for 600 participants to undergo 1:1 randomization. Only 155 patients were registered, with just 65 randomized [17]. Notably, many patients declined randomization due to a personal preference for undergoing PM. Given that this was a multicenter RCT involving 24 centers and failed to meet recruitment goals, the feasibility of conducting similar trials in other primary tumor types, such as malignant melanoma, is highly unlikely.

### Pulmonary metastasectomy or non-surgical therapy?

Metastatic melanoma had a grim prognosis before the advent of effective targeted immunotherapies. For decades, the only treatment option was dacarbazine alone or in combination, with 5-year survival rates ranging from 5 to 26% [18, 19]. The recent resurgence in effective treatments for stage IV melanoma has been driven by the introduction of immune checkpoint inhibitors targeting CTLA-4 and PD-1, as well as novel targeted therapies aimed at BRAF and MEK mutations. Nivolumab and pembrolizumab are the two approved anti-PD-1 drugs for use in metastatic melanoma and many other malignancies, such as lung cancer [20, 21]. Ipilimumab is the only approved anti-CTLA-4 drug.

Combining Ipilimumab with nivolumab yielded the best results, with 5-year survival rates of up to 60% [11, 22]. The combination is associated with more side effects, which can range from mild skin manifestations to life-threatening complications like colitis, neutropenia, agranulocytosis, and cardiac dysfunction [4, 23]. In our cohort, about 18.4% of patients stopped their therapy due to side effects, 21.5% of those were receiving Ipilimumab + Nivolumab.

Finally, patients with BRAF or MEK mutations who received targeted therapy showed a five-year survival rate of 71% [22, 23]. In our cohort, we performed a subgroup analysis of BRAF status, revealing that 55.3% of the non-surgical therapy group had a BRAF mutation. In contrast, only 35.4% of patients in the surgical therapy group had a BRAF mutation. The two groups showed similar survival rates with a log-rank of 0.48 (Fig. 3a). However, targeted therapy induces resistance through several pathways, including BRAF gene amplifications and MEK mutations [4, 24].

Patients with wild-type BRAF or those who have developed resistance have limited treatment options [25, 26]. We demonstrated that patients undergoing PM with wild-type BRAF showed significantly better survival rates than those receiving non-surgical therapy, with a log-rank of 0.02 (Fig. 3b). Therefore, patients with BRAF wild-type tumors should be considered for pulmonary metastasectomy, while those with BRAF-mutant disease may derive greater benefit from less invasive targeted systemic therapy. Most studies dealing with metastasectomy show worse survival rates than our cohort. He et al. included spinal and cerebral metastases and showed survival rates of < 1 year [27]. Hanna et al. included 99 patients with melanoma and pulmonary metastases. They showed 5-year survival rates of 21%. 23% of those received a lobectomy and > 60% a thoracotomy. Furthermore, in more than 12% of operations, the surgeons did not achieve an R0 resection. Lastly, they do not provide information on whether patients received surgery alone or as part of a multimodal approach [6]. We did not perform an extended pulmonary resection because there is no survival advantage to anatomical resection as PM. The majority received a VATS wedge resection. R0 resection was achieved in all cases. And 36% of patients received perioperative immunotherapy. Viehoff et al. showed that the patients benefit particularly from PM if nodules are < 20 mm. With a mean follow-up of 30.8 months, with a two-year survival of 54% [3].In our cohort, the mean lesion size in both groups was < 16 mm.

Our cohort's exceptionally high survival rates are likely due to improved therapy for the primary melanoma, more accurate diagnostics for pulmonary metastases, and more selective patient selection for PM compared to older studies [7, 8, 10].

### Which therapy for which patient?

Patients undergoing pulmonary metastasectomy (PM) demonstrated significantly better survival rates compared to those receiving non-surgical therapy. However, it is important to note that these patients are carefully selected. PM should only be considered when a complete (R0) resection is achievable. Additional prerequisites include curative treatment of the primary melanoma, absence of other distant metastases, and adequate pulmonary function [3, 9].

In our cohort, patients with BRAF wild-type status particularly benefited from PM. On the other hand, given the comparable survival rates, patients with BRAF mutations should primarily receive targeted therapy with BRAF inhibitors. Pulmonary metastasectomy should probably be reserved for cases in which systemic therapy fails. We also observed that patients undergoing non-surgical therapy had significantly more pulmonary metastases than those treated with PM. This can be attributed, in part, to the fact that patients with more than three pulmonary metastases are often not referred to thoracic surgeons due to the extent of disease.

Video-assisted thoracoscopic surgery (VATS) wedge resection is the gold standard for PM, whereas thoracotomy should be avoided due to its invasiveness. Patients with bilateral pulmonary metastases may be candidates for a two-stage surgical approach.

To ensure comparability, we only included patients with pulmonary metastases in both groups. Patients deemed unsuitable for surgery should be considered for non-surgical options such as radiotherapy or stereotactic body radiation therapy. In cases where PM is not feasible—particularly in patients with more than five pulmonary or distant metastases—treatment with checkpoint inhibitors, immunotherapy, or chemotherapy is recommended.

## Conclusion

The present study demonstrates that patients with metastatic malignant melanoma selected for pulmonary metastasectomy exhibit favorable survival outcomes compared to those receiving non-surgical therapy. In subgroup analyses, patients with BRAF-mutant tumors showed survival rates comparable to those undergoing pulmonary metastasectomy. Based on these findings, patients with BRAF wild-type tumors should be considered for pulmonary metastasectomy, whereas those with BRAF-mutant disease may derive greater benefit from upfront, less invasive targeted systemic therapy. Furthermore, pulmonary metastasectomy should be considered only when complete (R0) resection is achievable and when extrathoracic metastases have been excluded. Given the limited feasibility of conducting randomized controlled trials in this setting, the present study may contribute to the further establishment of pulmonary metastasectomy as a treatment option.

## Limitations

This study is a retrospective, single-center analysis and therefore entails the inherent limitations associated with retrospective designs, including the use of postoperative pathology data. Another limitation of our study is the relatively small sample size, which may limit the generalizability of the findings. Therefor more multicenter investigations are warranted to better elucidate the impact of pulmonary metastasectomy on the prognosis of patients with melanoma. By applying stringent inclusion and exclusion criteria, we aimed to establish a highly comparable cohort. Nevertheless, our results may still be influenced by confounding factors, which are inevitable when patients undergo different therapeutic approaches. Another limitation is that the non-surgical group predominantly received immune and targeted therapies, representing a more intensive systemic treatment. Consequently, differences in treatment intensity between groups must be acknowledged.

## Data Availability

The data underlying this article will be shared on reasonable request to the corresponding author.
